# Supplementary material for: Genetic regulation of methylation across East Asian and European populations
Source: Nat Commun. 2026 Feb 11;17:2616. doi: 10.1038/s41467-026-69372-6 (PMC13002944; doi:10.1038/s41467-026-69372-6)
Supplement: Supplementary file 1 — Supplementary Information [file 41467_2026_69372_MOESM1_ESM.pdf]

Genetic regulation of methylation across East Asian and European populations

Supplementary Figures

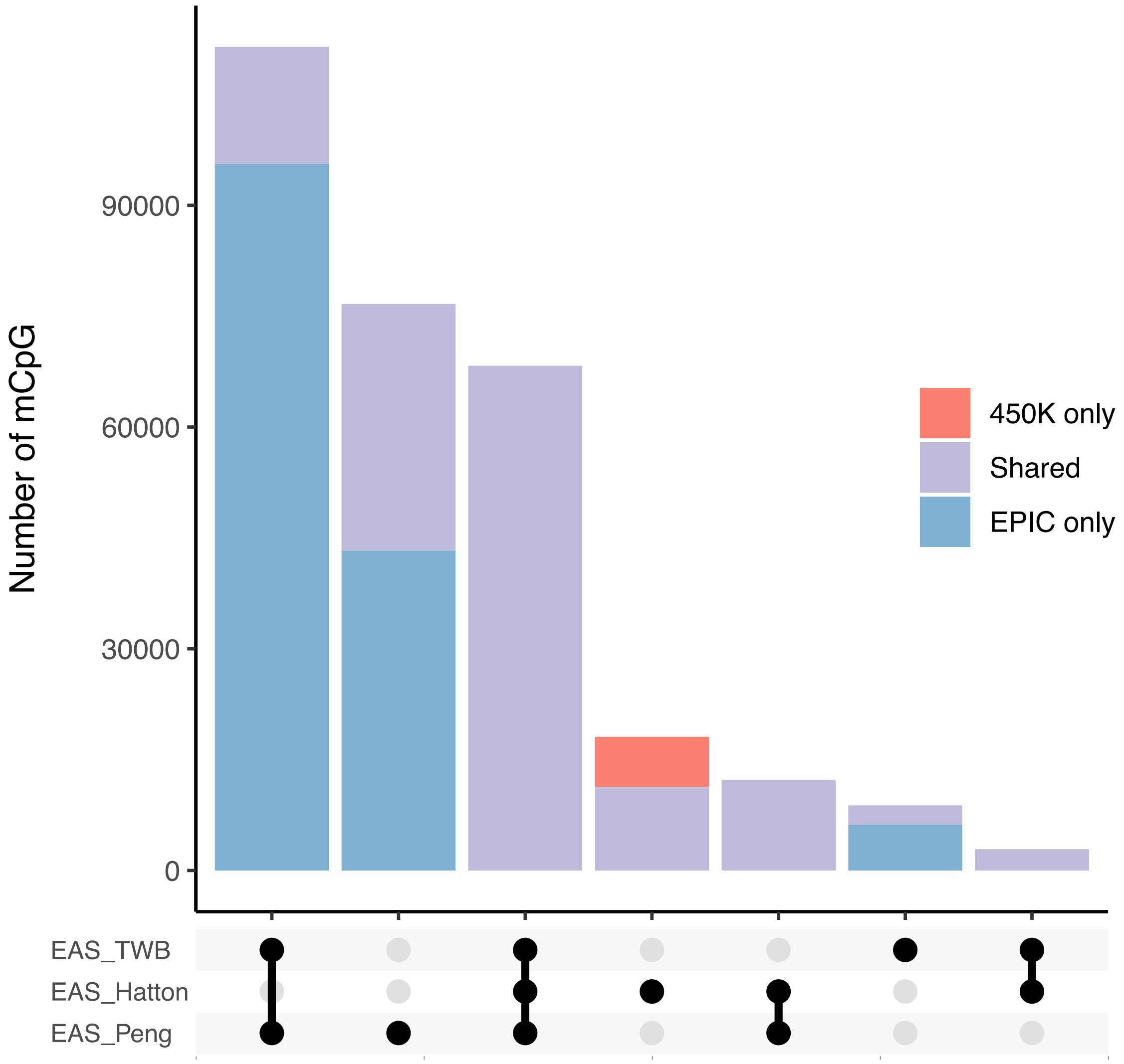

Supplementary Figure 1. Comparison of mCpG across EAS studies. Colors represent the source of CpG. 450K only: Probes for CpG are only designed in 450K array; Shared: Probes for CpG are designed in both 450K and EPIC arrays; EPIC only: Probes for CpG set are only designed in EPIC array. Source data are provided as a Source Data file.

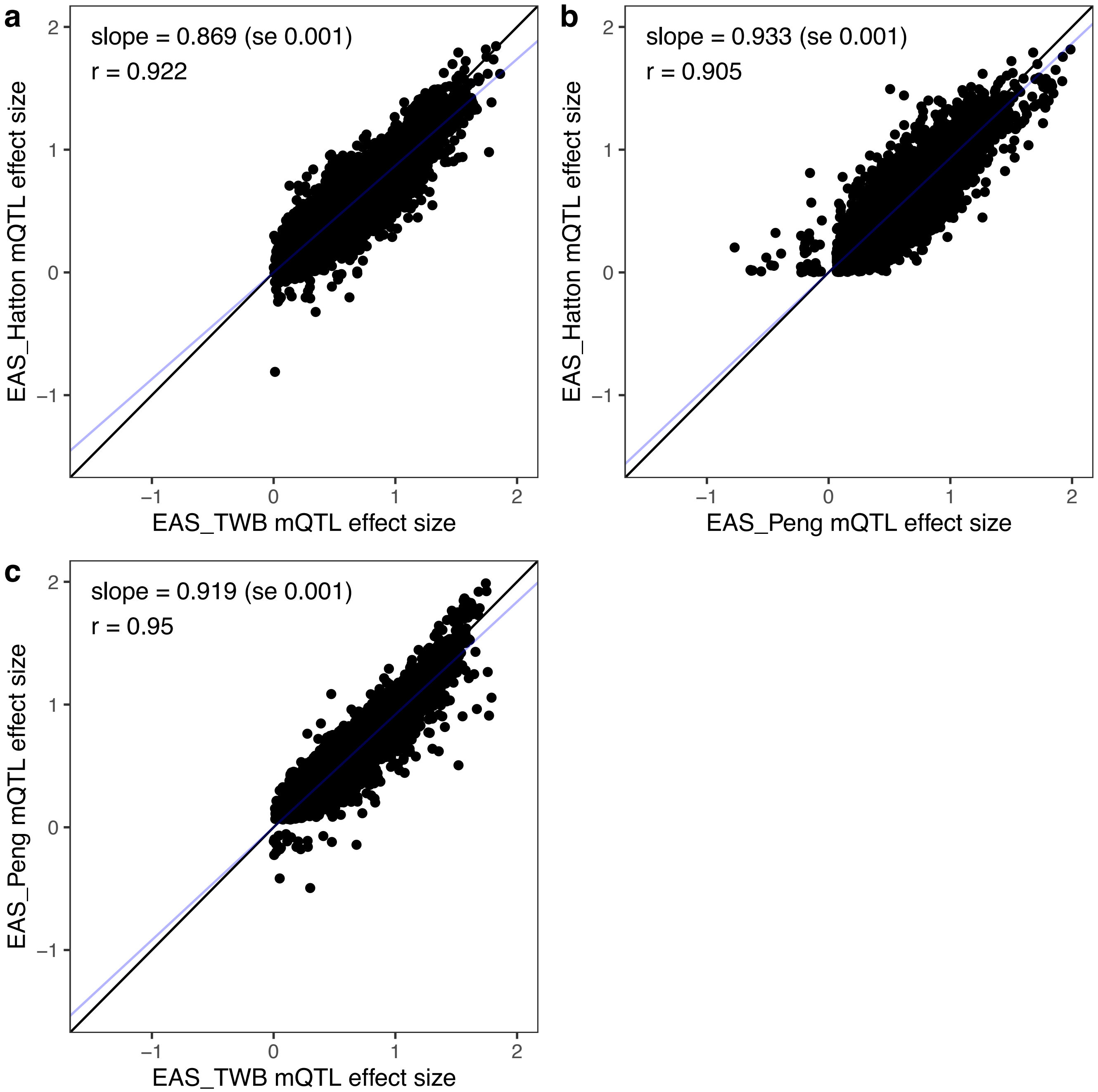

Supplementary Figure 2. Comparison of mQTL effect sizes across EAS studies. a, EAS_TWB vs. EAS_Hatton. **b**, EAS_Peng vs. EAS_Hatton. **c**, EAS_TWB vs. EAS_Peng. The comparisons include 20,926 mQTLs selected from the South Asian mQTL study as a benchmark (n=20,926). Black line: the diagonal line. Light blue line: the linear regression fitted line. slope: the slope of the linear regression. se: the standard error of the slope. r: the Pearson correlation coefficient. Source data are provided as a Source Data file.

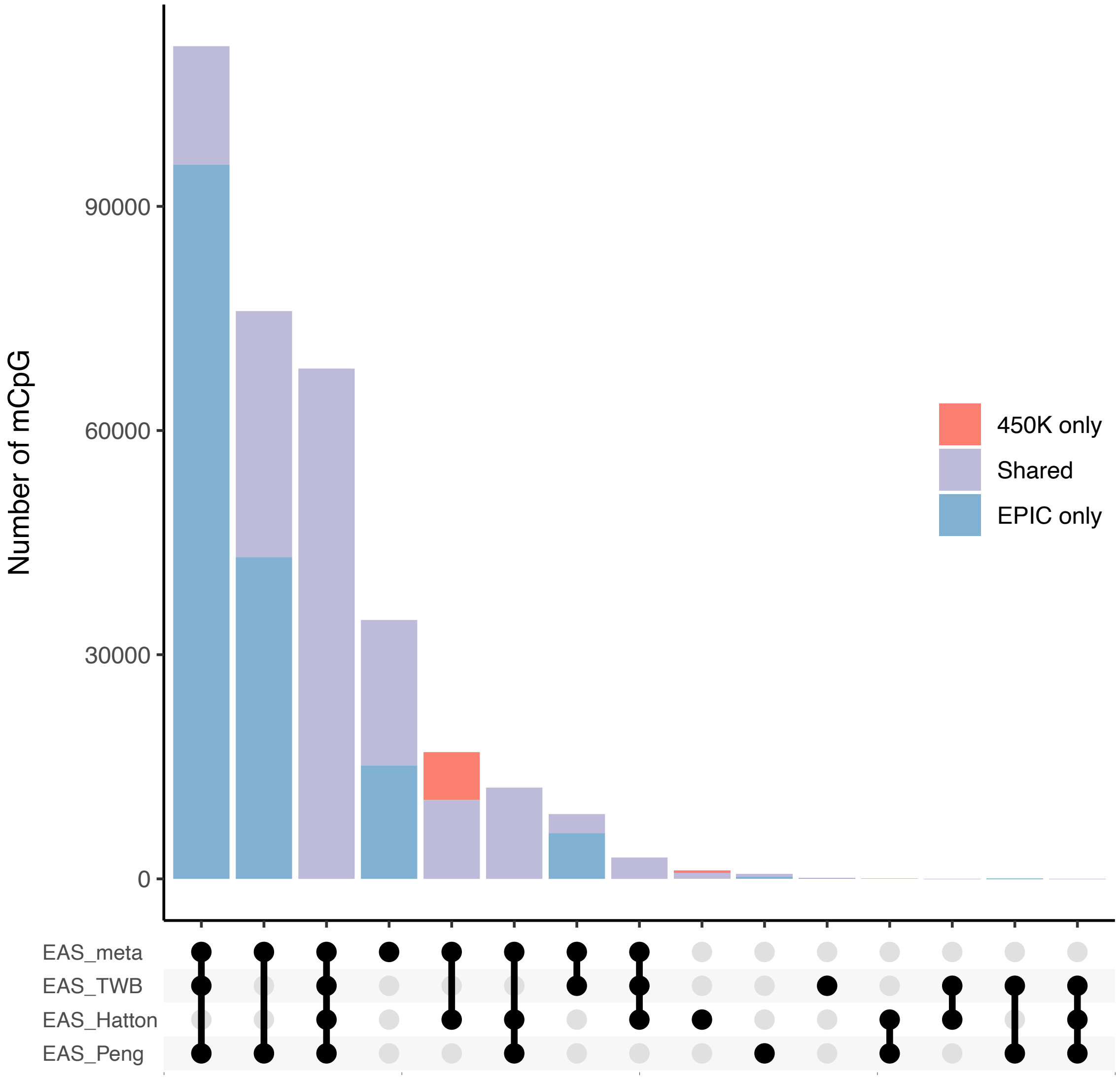

Supplementary Figure 3. Comparison of mCpG between EAS_meta and EAS studies. Colors represent the source of CpG. 450K only: Probes for CpG are only designed in 450K array; Shared: Probes for CpG are designed in both 450K and EPIC arrays; EPIC only: Probes for CpG set are only designed in EPIC array. Source data are provided as a Source Data file.

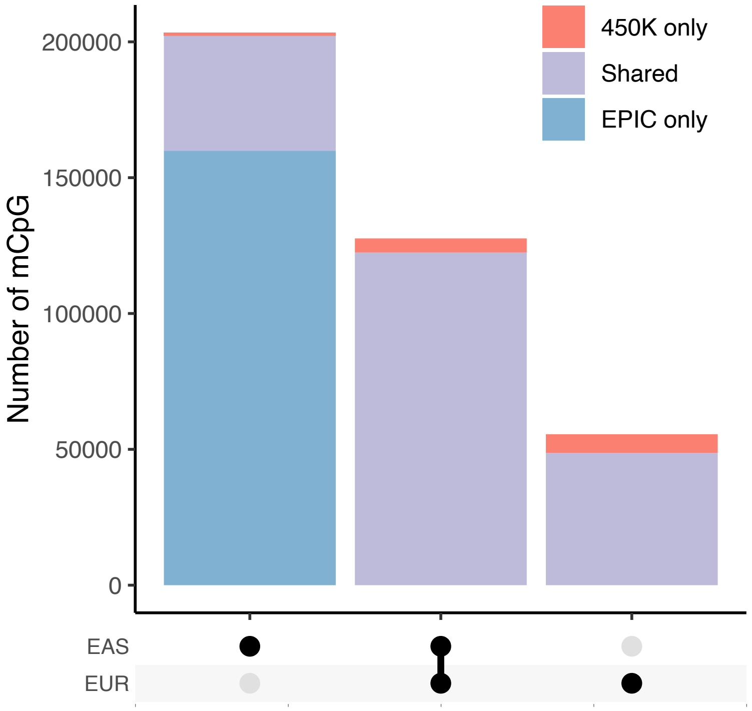

Supplementary Figure 4. Comparison of mCpG between EAS and EUR. Colors represent the source of CpG. 450K only: Probes for CpG are only designed in 450K array; Shared: Probes for CpG are designed in both 450K and EPIC arrays; EPIC only: Probes for CpG set are only designed in EPIC array. Source data are provided as a Source Data file.

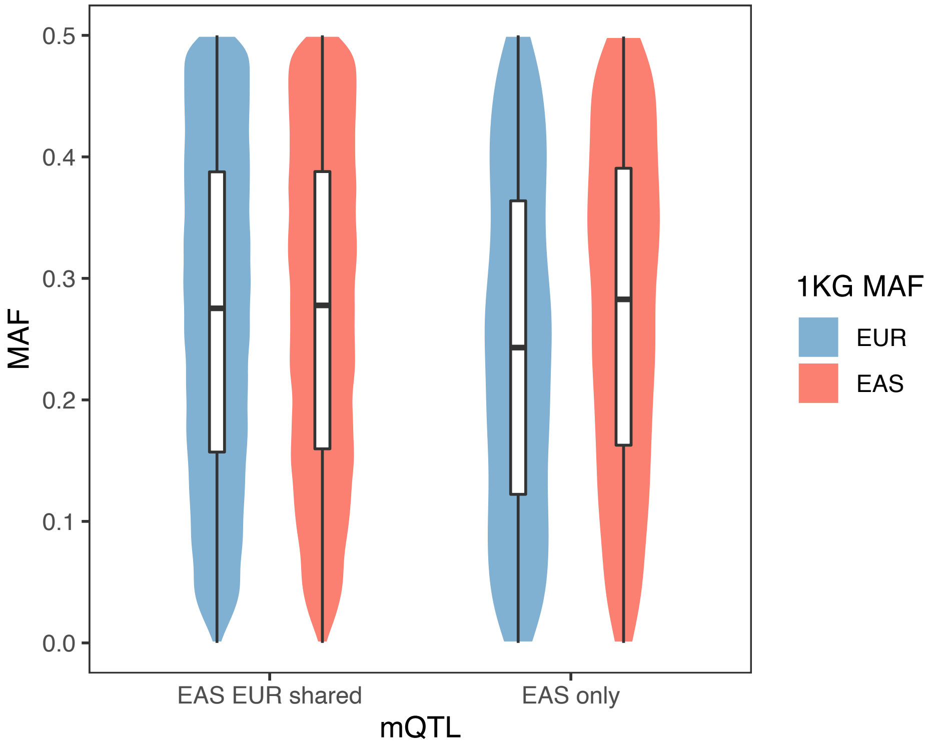

Supplementary Figure 5. Comparison of MAF of mQTL between EAS and EUR. EAS EUR shared: mQTLs shared in both EAS and EUR (n=95,321). EAS only: mQTLs are only in EAS (n=1,074). The MAF of 1KGP3 EAS and EUR of the index variants were presented for comparison. In the boxplot, center line: median; box limits: upper and lower 25% quartiles; whiskers: 1.5x interquartile range; point: outliers. Source data are provided as a Source Data file.

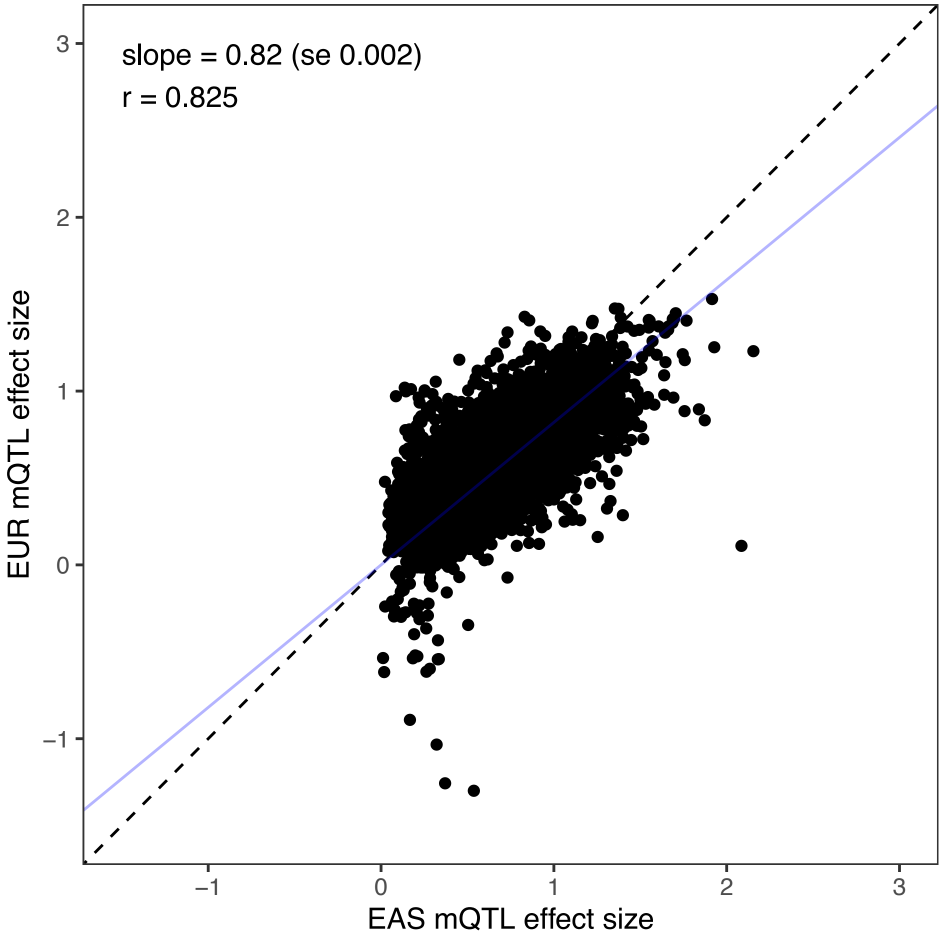

Supplementary Figure 6. Comparison of the effect size of mQTL across EAS and EUR. The comparison included 20,254 mQTLs selected from the South Asian mQTL study as a benchmark (n=20,254). The effect sizes of EAS and EUR are from EAS_meta and EUR_Min, respectively. Black dashed line: the diagonal line. Light blue line: the linear regression. slope: the slope of the linear regression. se: the standard error of slope. r: the Pearson correlation coefficient. Source data are provided as a Source Data file.

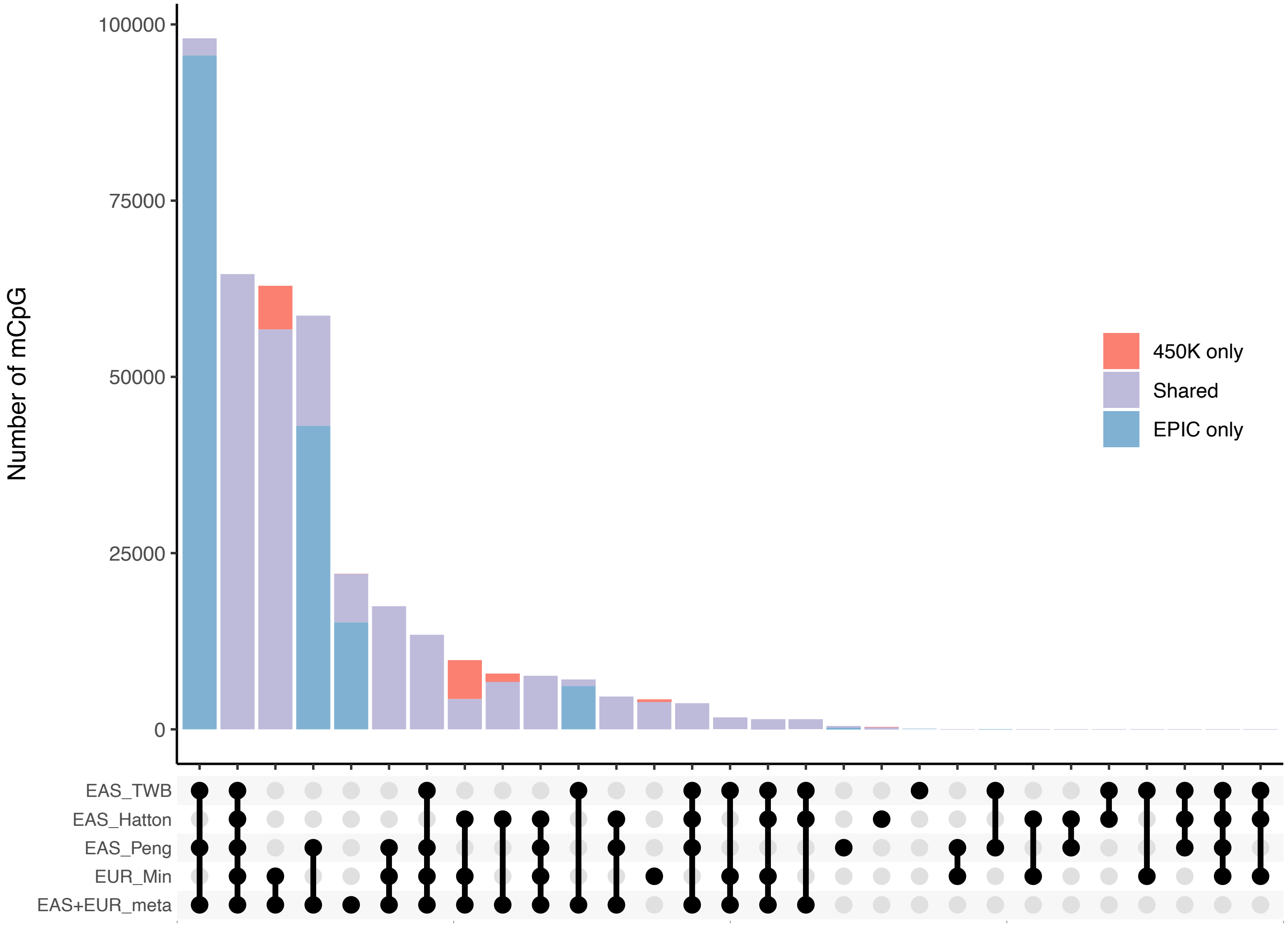

Supplementary Figure 7. Comparison of mCpG across meta-analysis, EUR, and EAS studies. Colors represent the source of CpG. EAS+EUR_meta: the EAS and EUR meta-analysis; 450K only: Probes for CpG are only designed in 450K array; Shared: Probes for CpG are designed in both 450K and EPIC arrays; EPIC only: Probes for CpG set are only designed in EPIC array. Source data are provided as a Source Data file.

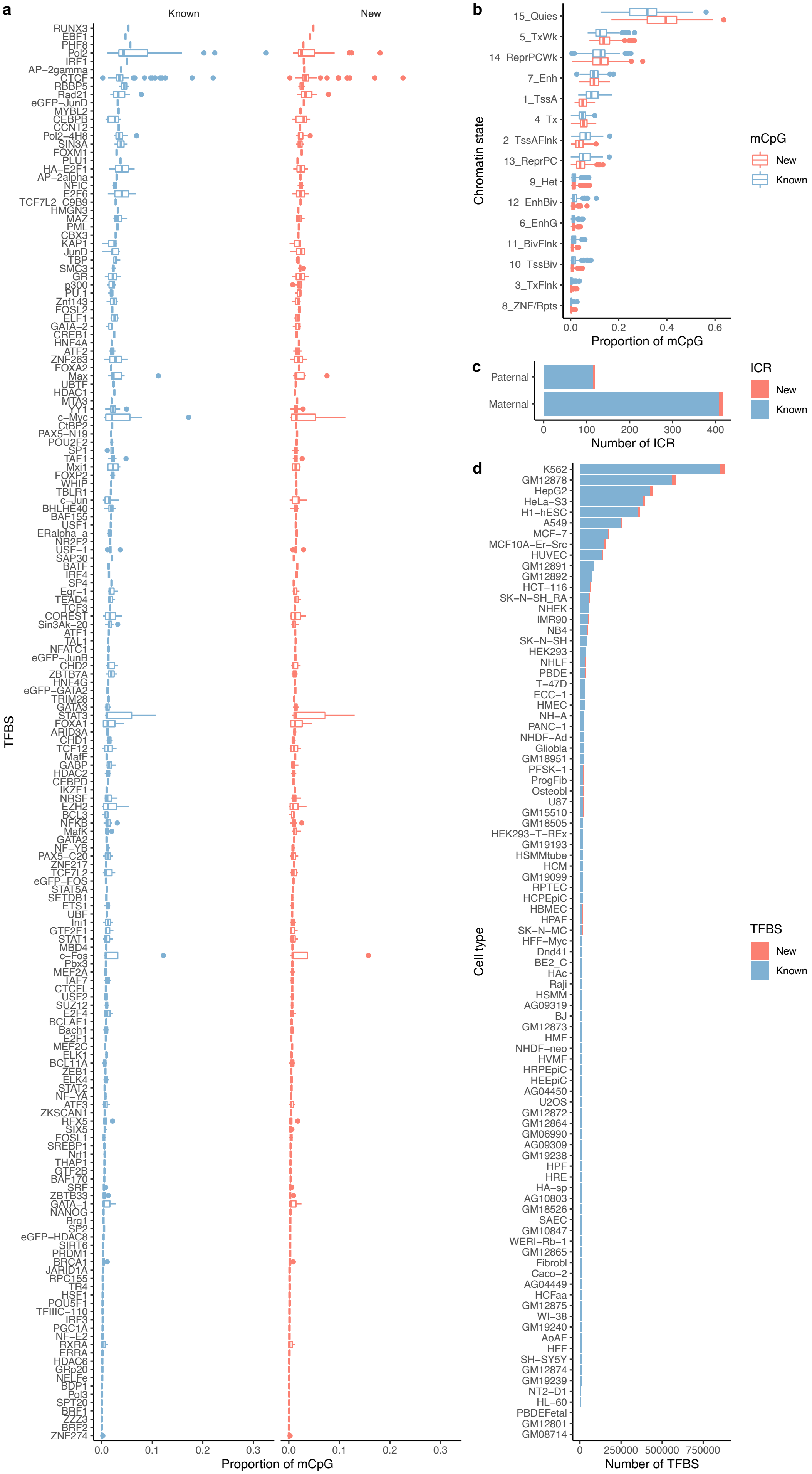
a
Known
New
RUNX3
EBF1
PHF8
Pol2
IRF1
AP−2gamma
CTCF
RBBP5
Rad21
eGFP−JunD
MYBL2
CEBPB
CCNT2
Pol2−4H8
SIN3A
FOXM1
PLU1
HA−E2F1
AP−2alpha
NFIC
E2F6
TCF7L2_C9B9
HMGN3
MAZ
PML
CBX3
KAP1
JunD
TBP
SMC3
GR
p300
PU.1
Znf143
FOSL2
ELF1
GATA−2
CREB1
HNF4A
ATF2
ZNF263
FOXA2
Max
UBTF
HDAC1
MTA3
YY1
c−Myc
CtBP2
PAX5−N19
POU2F2
SP1
TAF1
Mxi1
FOXP2
WHIP
TBLR1
c−Jun
BHLHE40
BAF155
USF1
ERalpha_a
NR2F2
USF−1
SAP30
BATF
IRF4
SP4
Egr−1
TEAD4
TCF3
COREST
Sin3Ak−20
ATF1
TAL1
NFATC1
eGFP−JunB
CHD2
ZBTB7A
HNF4G
eGFP−GATA2
TRIM28
GATA3
STAT3
FOXA1
ARID3A
CHD1
TCF12
MafF
GABP
HDAC2
CEBPD
IKZF1
NRSF
EZH2
BCL3
NFKB
MafK
GATA2
NF−YB
PAX5−C20
ZNF217
TCF7L2
eGFP−FOS
STAT5A
SETDB1
ETS1
UBF
Ini1
GTF2F1
STAT1
MBD4
c−Fos
Pbx3
MEF2A
TAF7
CTCFL
USF2
SUZ12
E2F4
BCLAF1
Bach1
E2F1
MEF2C
ELK1
BCL11A
ZEB1
ELK4
STAT2
NF−YA
ATF3
ZKSCAN1
RFX5
SIX5
FOSL1
SREBP1
Nrf1
THAP1
GTF2B
BAF170
SRF
ZBTB33
GATA−1
NANOG
Brg1
SP2
eGFP−HDAC8
SIRT6
PRDM1
BRCA1
JARID1A
RPC155
TR4
HSF1
POU5F1
TFIIIC−110
IRF3
PGC1A
NF−E2
RXRA
ERRA
HDAC6
GRp20
NELFe
BDP1
Pol3
SPT20
BRF1
ZZZ3
BRF2
ZNF274
TFBS
0.0 0.1 0.2 0.3
0.0 0.1 0.2 0.3
Proportion of mCpG
b
15_Quies
5_TxWk
14_ReprPCWk
7_Enh
1_TssA
4_Tx
2_TssAFlnk
13_ReprPC
9_Het
12_EnhBiv
6_EnhG
11_BivFlnk
10_TssBiv
3_TxFlnk
8_ZNF/Rpts
Chromatin state
0.0 0.2 0.4 0.6
Proportion of mCpG
mCpG
New
Known
c
Paternal
Maternal
0 100 200 300 400
Number of ICR
ICR
New
Known
d
K562
GM12878
HepG2
HeLa−S3
H1−hESC
A549
MCF−7
MCF10A−Er−Src
HUVEC
GM12891
GM12892
HCT−116
SK−N−SH_RA
NHEK
IMR90
NB4
SK−N−SH
HEK293
NHLF
PBDE
T−47D
ECC−1
HMEC
NH−A
PANC−1
NHDF−Ad
Gliobla
GM18951
PFSK−1
ProgFib
Osteobl
U87
GM15510
GM18505
HEK293−T−REx
GM19193
HSMMtube
HCM
GM19099
RPTEC
HCPEpiC
HBMEC
HPAF
SK−N−MC
HFF−Myc
Dnd41
BE2_C
HAc
Raji
HSMM
AG09319
BJ
GM12873
HMF
NHDF−neo
HVMF
HRPEpiC
HEEpiC
AG04450
U2OS
GM12872
GM12864
GM06990
AG09309
GM19238
HPF
HRE
HA−sp
AG10803
GM18526
SAEC
GM10847
WERI−Rb−1
GM12865
Fibrobl
Caco−2
AG04449
HCFaa
GM12875
WI−38
GM19240
AoAF
HFF
SH−SY5Y
GM12874
GM19239
NT2−D1
HL−60
PBDEFetal
GM12801
GM08714
Cell type
0 250000 500000 750000
Number of TFBS
TFBS
New
Known

Supplementary Figure 8. Regulatory functions of novel mCpG sites. a, Proportion of known and new mCpG located within the transcription factor binding sites (TFBS) across 91 cell types from the ENCODE project (n=91 cell types). **b,** Proportion of known and new mCpG located within the chromatin states across 127 cell types from the Roadmap Epigenomics project (n=127 cell types). **c,** The number of maternal and paternal imprinting control regions (ICR) overlapping with mCpG. Known: ICRs previously found to overlap with mCpGs. New: ICRs newly identified as overlapping with novel mCpGs. **d,** The number of TFBS overlapping with mCpG across cell types. Known: TFBS previously found to overlap with mCpGs. New: TFBSs newly identified as overlapping with novel mCpGs. In the boxplot, center line: median; box limits: upper and lower 25% quartiles; whiskers: 1.5x interquartile range; point: outliers. Source data are provided as a Source Data file.

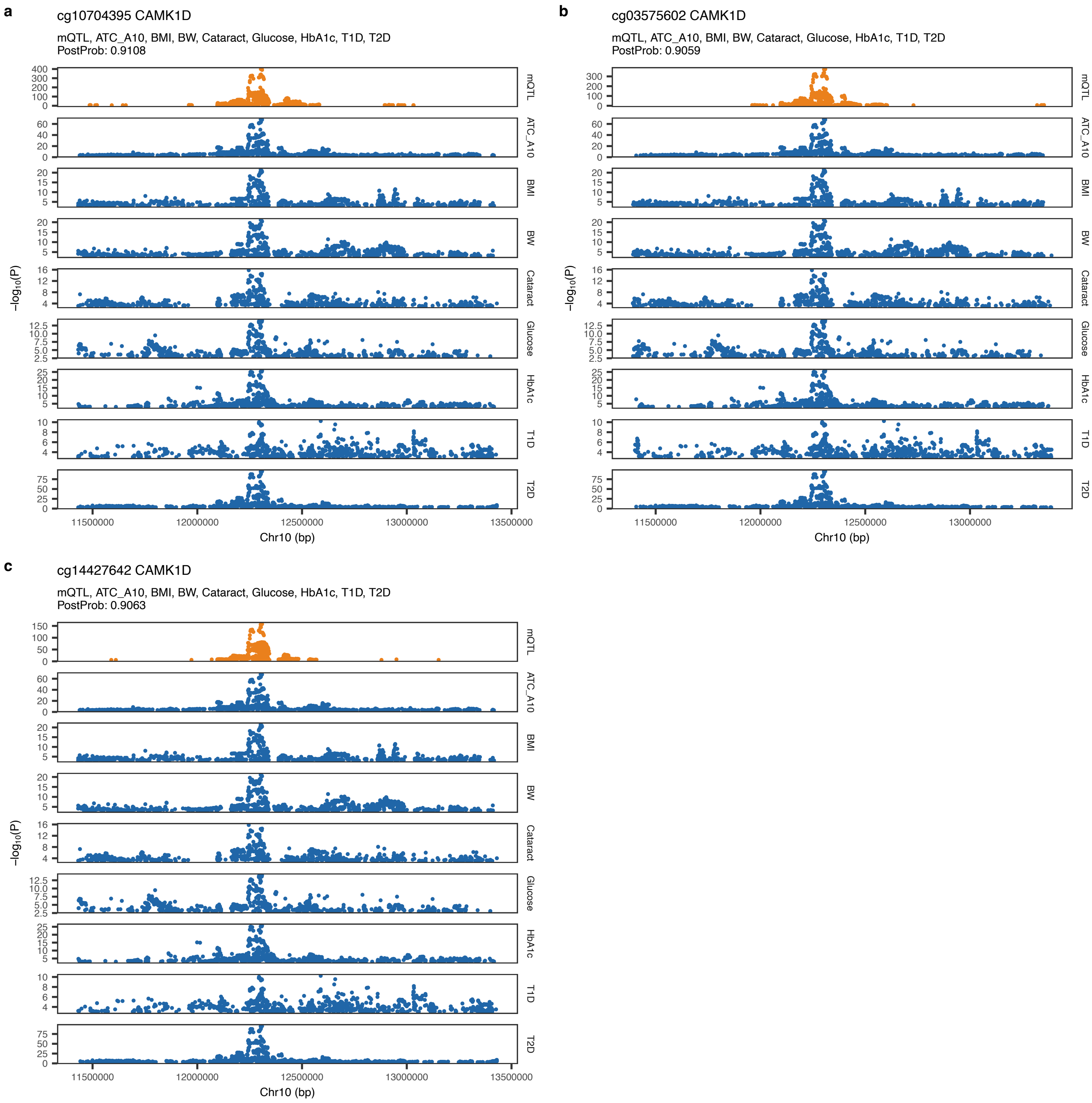

Supplementary Figure 9. Regional association plot of mCpGs cg10704395, cg03575602, and cg14427642. mCpGs cg10704395 (**a**), cg03575602 (**b**), and cg14427642 (**c**) colocalized with diabetes-related traits including drugs used in diabetes (ATC_A10), body mass index (BMI), birth weight (BW), cataract, glucose levels, Hemoglobin A1c (HbA1c), type 1 diabetes (T1D), and type 2 diabetes (T2D). The posterior probabilities of colocalization are 0.91, 0.90, and 0.90, respectively. The nearest gene of cg10704395, cg03575602, and cg14427642 is *CAMK1D*. Yellow: mQTL association; Blue: traits or diseases association. P: P-values originate from two-sided statistical tests within the EAS mQTL meta-analysis conducted in this study or from two-sided statistical tests within the GWAS performed in BBJ, without correction for multiple comparisons.

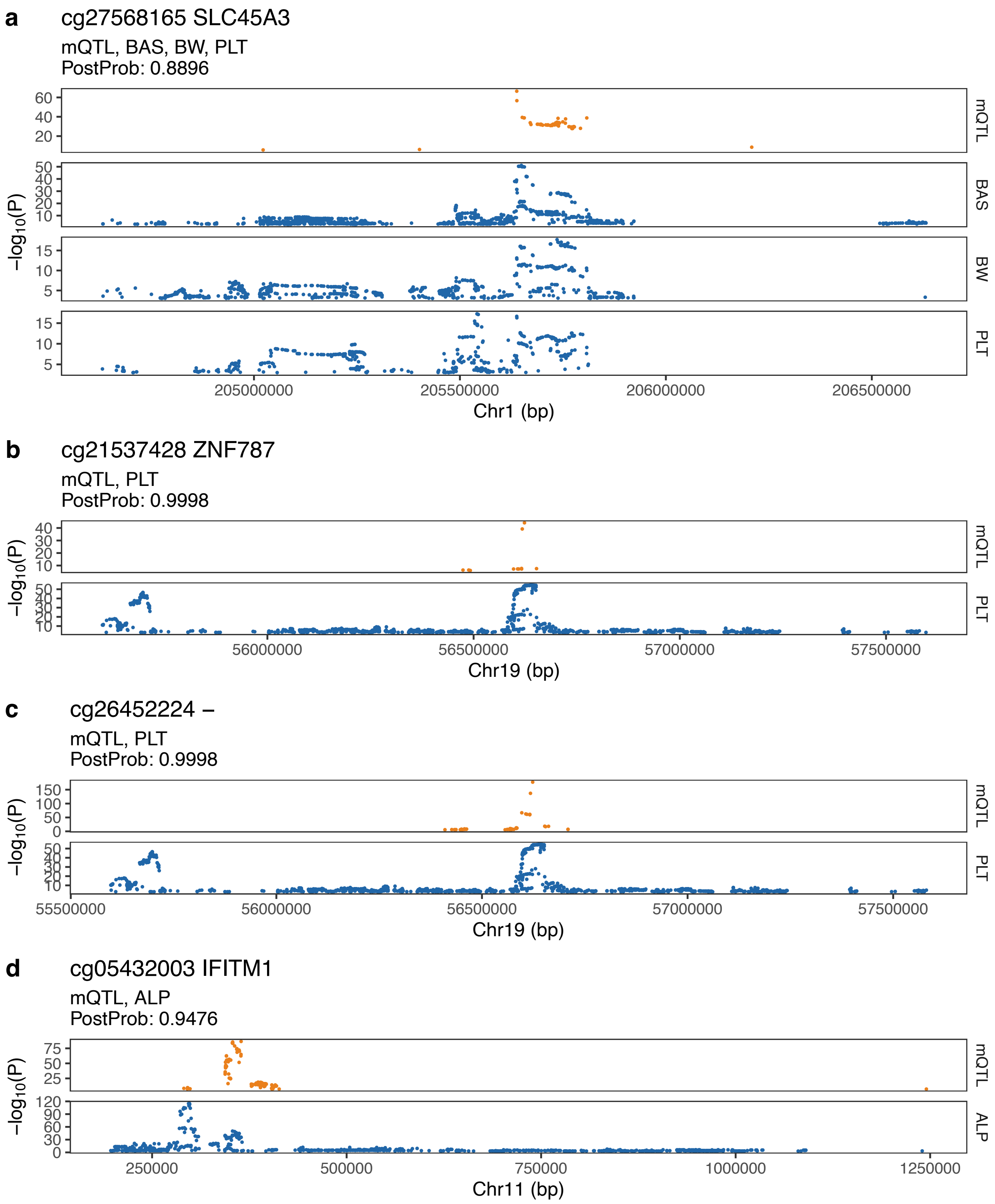

Supplementary Figure 10. Regional association plot of mCpGs identified only in EAS. a, cg27568165 colocalized with Basophil (BAS), Body weight (BW), and Platelet (PLT) with posterior probability = 0.88. The nearest gene of cg27568165 is *SLC45A3*. **b** and **c**, cg21537428 and cg26452224 colocalized with PLT with the posterior probability of colocalization = 0.99. The nearest gene of cg21537428 is *ZNF787*. **d**, cg05432003 colocalized with Alkaline phosphatase (ALP) with the posterior probability of colocalization = 0.94. The nearest gene of cg05432003 is *IFITM1*. P: P-values originate from two-sided statistical tests within the EAS mQTL meta-analysis conducted in this study or from two-sided statistical tests within the GWAS performed in BBJ, without correction for multiple comparisons.

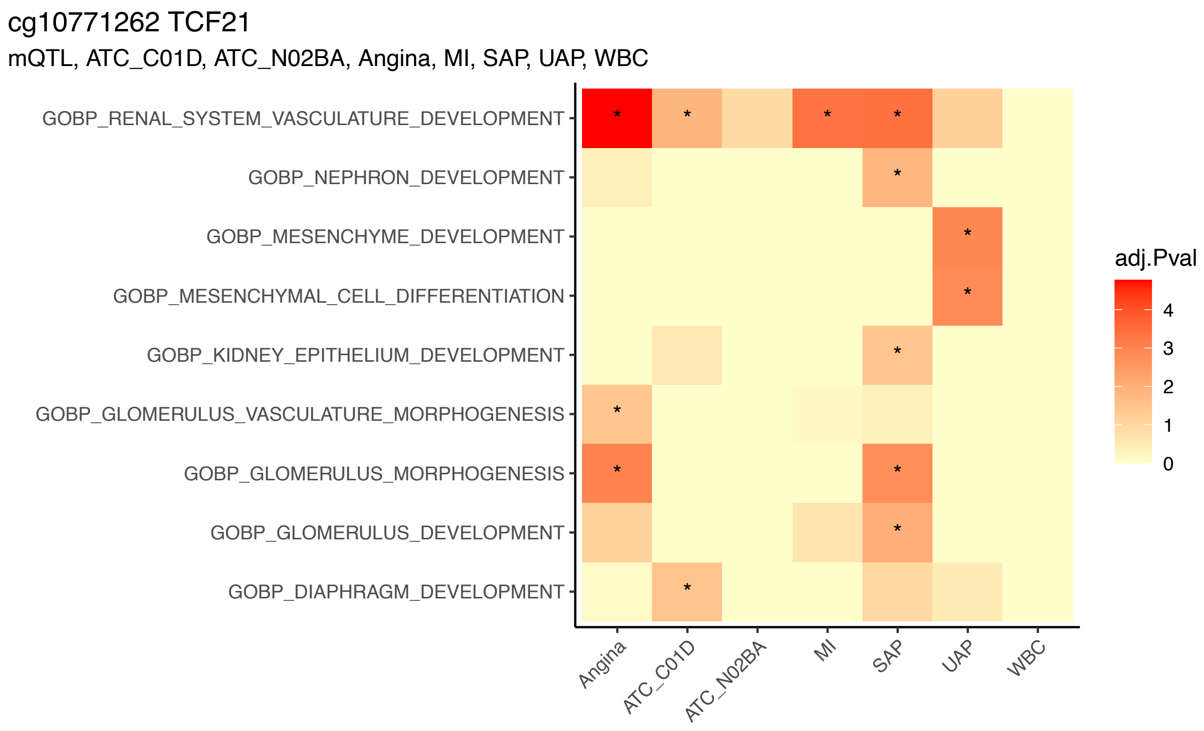

Supplementary Figure 11. Enrichment analysis of the GO biological process for traits co-localizing with mCpG cg10771262. mCpG cg10771262 exhibited pleiotropic effects on heart-related traits, including vasodilators used in cardiac diseases (ATC_C01D), salicylic acid derivatives (ATC_N02BA), angina pectoris (Angina), myocardial infarction (MI), stable angina pectoris (SAP), unstable angina pectoris (UAP), and white blood cell count (WBC). Its nearest gene, *TCF21*, likely mediates these effects. In this enrichment analysis, all the GO biological process gene sets containing *TCF21* were included. Only gene sets with at least one significantly enriched trait are displayed. adj.Pval: Bonferroni-corrected P-value from one-sided statistical tests within MAGMA. *: adj.Pval < 0.05.

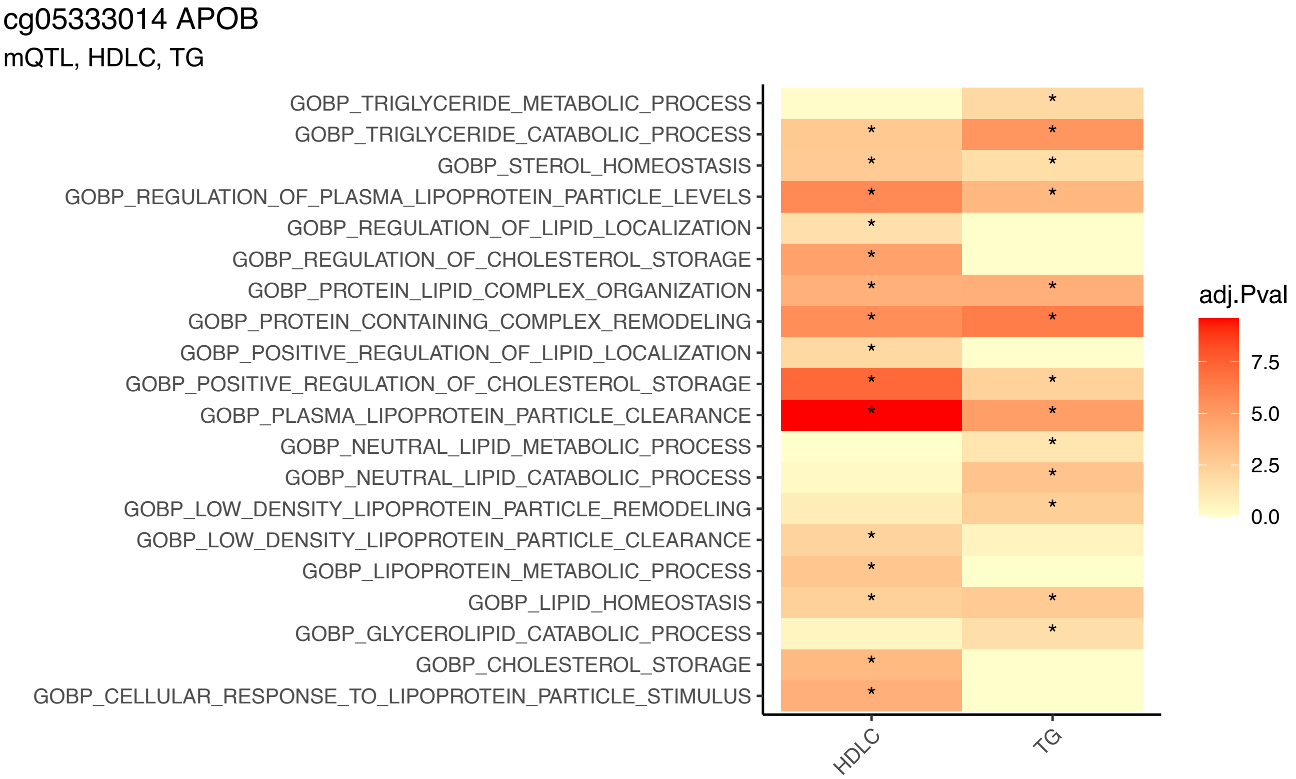

Supplementary Figure 12. Enrichment analysis of the GO biological process for traits co-localizing with mCpG cg05333014. mCpG cg05333014 exhibited pleiotropic effects on lipid-related traits, including high-density lipoprotein cholesterol (HDLC) and triglycerides (TG). Its nearest gene, *APOB*, likely mediates these effects. In this enrichment analysis, all the GO biological process gene sets containing *APOB* were included. Only gene sets with at least one significantly enriched trait are displayed. adj.Pval: Bonferroni-corrected P-value from one-sided statistical tests within MAGMA. *: adj.Pval < 0.05.

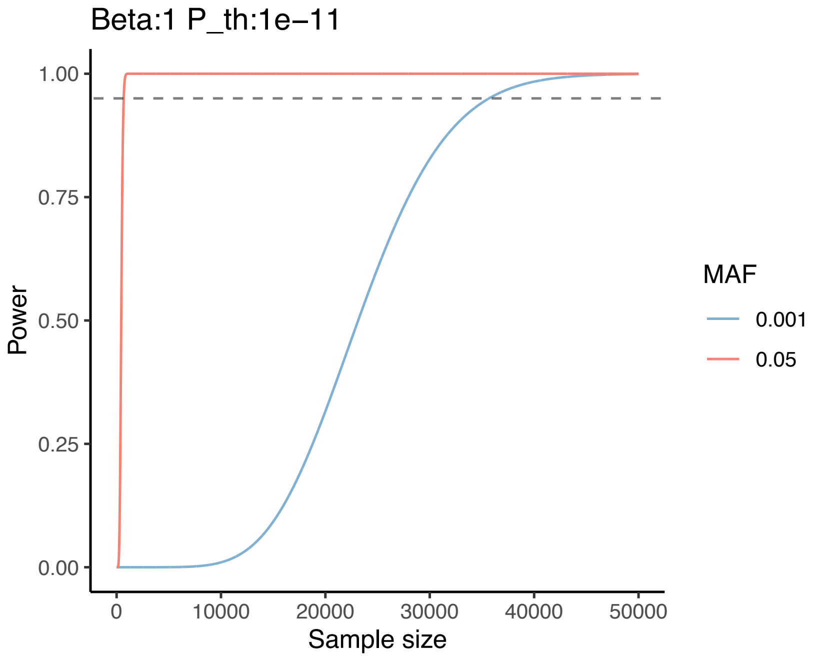

Supplementary Figure 13. Power for mQTL detection across MAF and sample size. The analysis assumes a variant with an effect size (Beta) of 1 standard deviation unit on methylation. Power: mQTL detection probability at a P-value threshold (P_th) of 1e-11 (two-sided). MAF: minor allele frequency. Dashed line: 95% power.
