# Supplementary material for: Genetic regulation of methylation across East Asian and European populations
Source: Nat Commun. 2026 Feb 11;17:2616. doi: 10.1038/s41467-026-69372-6 (PMC13002944; doi:10.1038/s41467-026-69372-6)
Supplement: Supplementary file 2 — Description of Additional Supplementary Files [file 41467_2026_69372_MOESM2_ESM.pdf]

Description of Additional Supplementary Files

Supplementary Data 1. Enrichment analysis of mCpG in CpG categories against non-mCpG

Supplementary Data 2. Enrichment analysis of mCpG in chromatin states against non-mCpG

Supplementary Data 3. SNP-heritability of BBJ traits with SNP-heritability > 0.03

Supplementary Data 4. Significant CpG-trait pairs in the HyPrColoc analysis

Supplementary Data 5. CpG-trait pairs in the SMR analysis

Supplementary Data 6. Significant CpG-trait pairs shared between SMR and HyPrColoc analyses

Supplementary Data 7. Gene set enrichment analysis for mCpG pleiotropic signals
