# Supplementary material for: Genetic regulation of methylation across East Asian and European populations
Source: Nat Commun. 2026 Feb 11;17:2616. doi: 10.1038/s41467-026-69372-6 (PMC13002944; doi:10.1038/s41467-026-69372-6)
Supplement: Supplementary file 4 — Reporting Summary [file 41467_2026_69372_MOESM4_ESM.pdf]

nature portfolio

Corresponding author(s): Chia-Yen Chen, Yen-Chen Anne Feng, Hailiang Huang, Yen-Feng Lin

Last updated by author(s): Dec 10, 2025

Reporting Summary

Nature Portfolio wishes to improve the reproducibility of the work that we publish. This form provides structure for consistency and transparency in reporting. For further information on Nature Portfolio policies, see our Editorial Policies and the Editorial Policy Checklist.

Statistics

For all statistical analyses, confirm that the following items are present in the figure legend, table legend, main text, or Methods section.

n/a	Confirmed	
☐	☒	The exact sample size (*n*) for each experimental group/condition, given as a discrete number and unit of measurement
☐	☒	A statement on whether measurements were taken from distinct samples or whether the same sample was measured repeatedly
☐	☒	The statistical test(s) used AND whether they are one- or two-sided<br>*Only common tests should be described solely by name; describe more complex techniques in the Methods section.*
☐	☒	A description of all covariates tested
☐	☒	A description of any assumptions or corrections, such as tests of normality and adjustment for multiple comparisons
☐	☒	A full description of the statistical parameters including central tendency (e.g. means) or other basic estimates (e.g. regression coefficient) AND variation (e.g. standard deviation) or associated estimates of uncertainty (e.g. confidence intervals)
☐	☒	For null hypothesis testing, the test statistic (e.g. *F*, *t*, *r*) with confidence intervals, effect sizes, degrees of freedom and *P* value noted<br>*Give P values as exact values whenever suitable.*
☒	☐	For Bayesian analysis, information on the choice of priors and Markov chain Monte Carlo settings
☒	☐	For hierarchical and complex designs, identification of the appropriate level for tests and full reporting of outcomes
☐	☒	Estimates of effect sizes (e.g. Cohen's *d*, Pearson's *r*), indicating how they were calculated

Our web collection on statistics for biologists contains articles on many of the points above.

Software and code

Policy information about availability of computer code

Data collection	No software was used for data collection.
Data analysis	Computer code relating to this study includes:<br>plink 1. 9beta: https://www.cog-genomics.org/plink/<br>Eagle v2.4: https://alkesgroup.broadinstitute.org/Eagle/<br>Minimac4: https://genome.sph.umich.edu/wiki/Minimac4<br>QTLtools 1.3.1: https://qtltools.github.io/qtltools/<br>chAMP: https://bioconductor.org/packages/release/bioc/html/ChAMP.html<br>PEER: https://www.sanger.ac.uk/tool/peer/<br>LDSC v1.0.1: https://github.com/bulik/ldsc<br>METAL v2011-03-25: https://genome.sph.umich.edu/wiki/METAL<br>Coloc v5 : https://chr1swallace.github.io/coloc/<br>HyPrColoc 0.0.2: https://github.com/jrs95/hyprcoloc<br>SMR: https://yanglab.westlake.edu.cn/software/smr/<br>MAGMA v1.10: https://cncr.nl/research/magma/

For manuscripts utilizing custom algorithms or software that are central to the research but not yet described in published literature, software must be made available to editors and reviewers. We strongly encourage code deposition in a community repository (e.g. GitHub). See the Nature Portfolio guidelines for submitting code & software for further information.

Data

Policy information about availability of data

All manuscripts must include a data availability statement. This statement should provide the following information, where applicable:

- Accession codes, unique identifiers, or web links for publicly available datasets
- A description of any restrictions on data availability
- For clinical datasets or third party data, please ensure that the statement adheres to our policy

Data from the TWB are available at https://www.biobank.org.tw/english.php; mQTL Summary statistics for Taiwan biobank (EAS_TWB), EAS_meta and EAS +EUR_meta are available at https://lin-lab.site/data/; mQTL summary statistics from EAS_Hatton are available at https://yanglab.westlake.edu.cn/software/smr/#mQTLsummarydata ; mQTL summary statistics from EAS_Peng are available at https://www.biosino.org/node/project/detail/OEP002902; mQTL summary statistics from EUR_Min are available at http://mqtldb.godmc.org.uk/downloads; mQTL summary statistics from South Asian mQTL study are available at https://zenodo.org/record/5196216#.YRZ3TfJxeUk; GWAS summary statistics for 220 traits from Biobank Japan are available at https://pheweb.jp/downloads; GWAS summary statistics for IBD in EAS are available at https://www.ibdgenetics.org; GWAS summary statistics for SCZ in EAS are available at https://pgc.unc.edu/for-researchers/download-results/; 1000 Genomes Project Phase 3 is available from https://www.internationalgenome.org/category/phase-3/; CpG annotation is available from https://zhouserver.research.chop.edu/InfiniumAnnotation/20180909/EPIC/EPIC.hg19.manifest.tsv.gz; CpG categories annotation is available from https://support.illumina.com/downloads/infinium-methylationepic-v1-0-product-files.html; Chromatin, Transcription factor binding sites, Imprinting control regions, and CTCF binding sites annotation data for CpG is available from http://zwdzwd.github.io/InfiniumAnnotation#current; GO gene sets from MSigDB v2025.1 are available at https://www.gsea-msigdb.org/gsea/msigdb

Research involving human participants, their data, or biological material

Policy information about studies with human participants or human data. See also policy information about sex, gender (identity/presentation), and sexual orientation and race, ethnicity and racism.

Reporting on sex and gender	At recruitment, participants provided a written informed consent and had their gender information collected through questionnaires. We inferred their genetic sex and excluded samples showing inconsistencies between genetic sex and self-reported sex. In mQTL analysis, we incorporated sex as a covariate into a linear regression model to adjust for its influence on methylation levels.
Reporting on race, ethnicity, or other socially relevant groupings	The TWB cohort represents a Han Chinese population and does not include data from indigenous tribes due to strict ethical and legal guidelines. Principal component analysis (PCA) was conducted using the 1000 Genomes Project phase 3 (1KGP3) data as a reference panel to infer genetic ancestry and identify population outliers. In mQTL analysis, we incorporated first 20 genetic PCs as covariates into a linear regression model to adjust for their influence on methylation levels.
Population characteristics	The TWB is a community-based cohort, focusing on individuals from Taiwan aged 20 to 70 with no prior cancer diagnosis. It represents a Han Chinese population and does not include data from indigenous tribes due to strict ethical and legal guidelines. The cohort consists of 64% females and 36% males.
Recruitment	Recruitment sites were strategically placed across Taiwan, taking into account the population density of various counties and cities. During the enrollment process, participants provided written informed consent and then underwent various assessments including questionnaires, physical examinations, and blood and urine tests. Baseline data collection was comprehensive, capturing a wide range of phenotypic measurements.
Ethics oversight	Written Informed consent and permission to share the data were obtained from all subjects, in compliance with the guidelines specified by the recruiting center's institutional review board.<br>This study has been approved by the Ethics and Governance Council (EGC) of Taiwan Biobank (TWBR10907-05) and the Institutional Review Board (IRB) of National Health Research Institutes, Taiwan (EC1090402-E).

Note that full information on the approval of the study protocol must also be provided in the manuscript.

Field-specific reporting

Please select the one below that is the best fit for your research. If you are not sure, read the appropriate sections before making your selection.

☒ Life sciences ☐ Behavioural & social sciences ☐ Ecological, evolutionary & environmental sciences

For a reference copy of the document with all sections, see nature.com/documents/nr-reporting-summary-flat.pdf

Life sciences study design

All studies must disclose on these points even when the disclosure is negative.

Sample size	Previous mQTL studies in EAS, conducted with sample sizes ranging from 2,000 to 3,500, exhibited robust statistical power. This study incorporated all available samples provided by collaborators.
Data exclusions	For genotype data, we excluded samples exhibiting a heterozygosity rate beyond 6 standard deviations from the sample mean, as well as samples showing inconsistencies between genetic sex and self-reported sex. Within the EAS population, variants with low call rates or failing

the Hardy-Weinberg equilibrium (HWE) test were discarded. In post-imputation QC, we removed variants with poor imputation quality (INFO score < 0.6) or low MAF (MAF < 0.005), yielding approximately 8 million genetic variants in the mQTL analysis.
For DNA methylation data, we used the chAMP pacakage to automatically filter out SNPs that might influence the analysis results. Then we filtered:1) probes with detection P-value > 0.01, 2) probes with less than 3 beads in at least 5% of the samples per probe; 3) all non-CpG probes; 4) all multi-hit probes.

Replication

The analysis was performed on four independent cohorts that serve the purpose of replication with each other.

Randomization

No randomization was conducted. The current study is a large-scale genetics study. Randomness is achieved from the nature of how alleles are distributed in the population. Alleles are randomly passed from parent to offspring during meiosis, hence the nature of these types of studies tend to be shielded from extraneous confounds of standard epidemiological studies. One exception of this is the population structure, which can confound the random passing of genetic alleles. To resolve the problem, we performed the principal component (PC) analysis to capture the population structure as PCs. We then included PCs in mQTL anlaysis as covariates to control the confounding driven by the population structure.

Blinding

The nature of the study is in of itself blind to study recruiters. No one would beforehand know the genotype and DNA methylation level of the sample collected in the current study.

Reporting for specific materials, systems and methods

We require information from authors about some types of materials, experimental systems and methods used in many studies. Here, indicate whether each material, system or method listed is relevant to your study. If you are not sure if a list item applies to your research, read the appropriate section before selecting a response.

Materials & experimental systems

n/a	Involved in the study	
☒	☐	Antibodies
☒	☐	Eukaryotic cell lines
☒	☐	Palaeontology and archaeology
☒	☐	Animals and other organisms
☒	☐	Clinical data
☒	☐	Dual use research of concern
☒	☐	Plants

Methods

n/a	Involved in the study	
☒	☐	ChIP-seq
☒	☐	Flow cytometry
☒	☐	MRI-based neuroimaging

Plants

Seed stocks

Report on the source of all seed stocks or other plant material used. If applicable, state the seed stock centre and catalogue number. If plant specimens were collected from the field, describe the collection location, date and sampling procedures.

Novel plant genotypes

Describe the methods by which all novel plant genotypes were produced. This includes those generated by transgenic approaches, gene editing, chemical/radiation-based mutagenesis and hybridization. For transgenic lines, describe the transformation method, the number of independent lines analyzed and the generation upon which experiments were performed. For gene-edited lines, describe the editor used, the endogenous sequence targeted for editing, the targeting guide RNA sequence (if applicable) and how the editor was applied.

Authentication

Describe any authentication procedures for each seed stock used or novel genotype generated. Describe any experiments used to assess the effect of a mutation and, where applicable, how potential secondary effects (e.g. second site T-DNA insertions, mosiacism, off-target gene editing) were examined.
